# Neuron-derived FGF10 ameliorates cerebral ischemia injury via inhibiting NF-κB-dependent neuroinflammation and activating PI3K/Akt survival signaling pathway in mice

**DOI:** 10.1038/srep19869

**Published:** 2016-01-27

**Authors:** Yong-Hua Li, Hai-Long Fu, Mou-Li Tian, Yong-Qiang Wang, Wei Chen, Lin-Lin Cai, Xu-Hui Zhou, Hong-Bin Yuan

**Affiliations:** 1 Department of Anesthesiology, Changzheng Hospital, Second Military Medical University, 415 Fengyang Road, Shanghai, 200003, China; 2 Department of Anesthesiology, Shuguang Hospital, Traditional Chinese Medicine University, Shanghai, 201203, China; 3 Department of Orthopedic Surgery, Changzheng Hospital, Second Military Medical University, 415 Fengyang Road, Shanghai, 200003, China

## Abstract

FGF10 is a member of fibroblast growth factors (FGFs). We previously showed that FGF10 protects neuron against oxygen-glucose deprivation injury *in vitro*; however, the effect of FGF10 in ischemic stroke *in vivo* is unknown. In the present study, we showed that FGF10 was mainly expressed in neurons but not astrocytes, and detected FGF10 in mouse cerebrospinal fluid. The FGF10 levels in neurons culture medium and cell lysate were much higher than those in astrocytes. FGF10 expression in brain tissue and FGF10 level in CSF were increased in mouse middle cerebral artery occlusion (MCAO) model. Administration of FGF10 into lateral cerebroventricle not only decreased MCAO-induced brain infarct volume and neurological deficit, but also reduced the number of TUNEL-positive cells and activities of Caspases. Moreover, FGF10 treatment depressed the triggered inflammatory factors (TNF-α and IL-6) and NF-κB signaling pathway, and increased phosphorylation of PI3K/Akt signaling pathway. Blockade of PI3K/Akt signaling pathway by wortmannin and Akt1/2-kinase inhibitor, partly compromised the neuroprotection of FGF10. However, blockade of PI3K/Akt signaling pathway did not impair the anti-inflammation action of FGF10. Collectively, our results demonstrate that neuron-derived FGF10 ameliorates cerebral ischemia injury via inhibiting NF-κB-dependent neuroinflammation and activating PI3K/Akt survival signaling pathway in mice.

Stroke is the third leading cause of death in the United States and results in substantial health-care expenditures^1^. In the past two decades, over 1000 clinical trials have failed to demonstrate a benefit in treating stroke, with the exception of thrombolytics^2^, suggesting that the physiopathological mechanisms of ischemic stroke are far more complex recognized previously. Besides the insufficient oxygen and glucose delivery, other detrimental factors such as excitotoxicity, acidotoxicity, nitrative stress and especially, post-ischemic neuroinflammation, contribute to the last outcome of ischemic stroke^3^. Moreover, the circulating factors released by brain or other peripheral organs may also affect the pathogenesis of stroke^4^,^5^.

Fibroblast growth factors (FGFs) are a family of growth factors that share a number of biochemical and biological properties released by various tissues^6^. FGFs are essential for embryonic development and often function postnatally in the response to injury and in the regulation of electrical excitability of cells. They regulate fundamental biological processes and play important roles in numerous diseases, including bone remodeling, cardiovascular diseases, metabolism regulation, kidney diseases and cancer^6^,^7^,^8^,^9^. FGF10 is a member of the FGFs family. It was firstly cloned from rat embryos in 1996^10^. Up to now, most studies about FGF10 focus on the role of FGF10 in development, differentiation and regeneration. FGF10 has been found to play an essential role in mesenchymal-epithelial interactions for the proper development of many organs including adipose, limb, lung and prostate^11^,^12^,^13^. FGF-10 also regulates cell mitogenesis, motility, differentiation and migration^14^. FGF10 critically regulates fibroblast development^15^. Nevertheless, the potential roles of FGF10 in other biological functions are poorly understood.

In our previous study^16^, we for the first time demonstrated that exogenous FGF10 administration prevented cultured cortical neurons from cell death caused by oxygen-glucose deprivation (OGD), an *in vitro* ischemic stroke model. However, the pathophysiological changes in ischemic brain are far more complex than that in neuron alone because astrocytes, microglia, inflammatory cells and even microvascular endothelial cells participate in this process^17^,^18^. Thus, we further investigated the potential role of FGF10 on ischemic stroke *in vivo* in this study. We found that FGF10 was mainly expressed in neurons and could be released from neurons. We also tested the effect of FGF10 in mice with mouse middle cerebral artery occlusion (MCAO), a widely-used *in vivo* ischemic stroke model. Our data indicate that intracranial FGF10 administration protected experimental ischemic stroke via activating PI3K/Akt pathway and suppressing neuroinflammation triggered by cerebral ischemia.

## Results

### Neuron is the main source of brain FGF10

**Figure 1:**
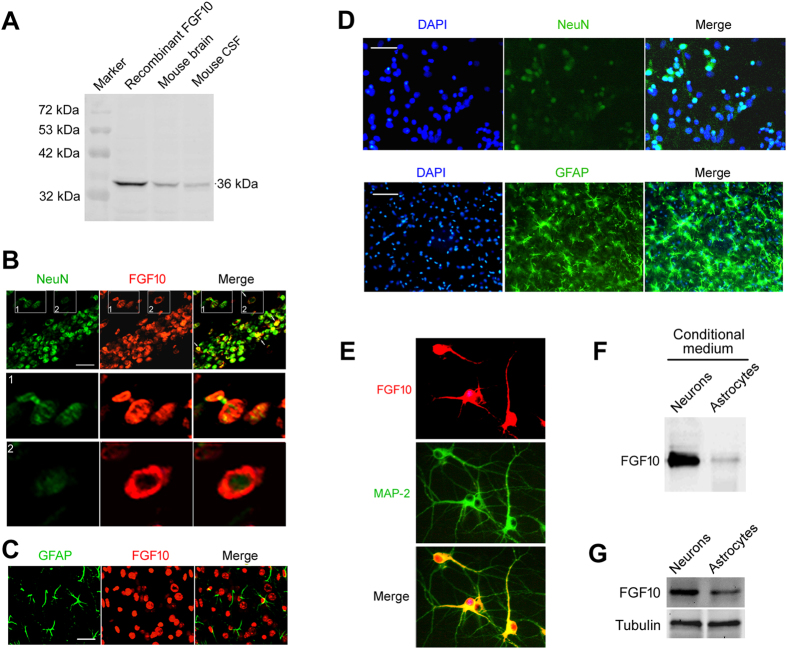
Neuron is the main source of brain FGF10. (**A**) FGF levels in mouse brain tissue (lane 2) and cerebrospinal fluid (CSF, lane 3) were determined by immunoblotting. Recombinant FGF10 (lane 1) was used as positive control. (**B**) FGF10 expression pattern in neurons (stained by NeuN) in cerebral cortex (frontal lobe) was determined by double immunohistochemical staining. The images in white boxes were further enlarged to show the intracellular distribution of FGF10. Scale bar, 50 μm. (**C**) FGF10 expression pattern in astrocytes (stained by GFAP) in cerebral cortex (frontal lobe) was determined by double staining immunohistochemistry. Scale bar, 50 μm. (**D**) Double staining immunohistochemistry showing the primary cultured neurons and astrocytes. DAPI was used to stain nuclei. Scale bar, 100 μm. (**E**) MAP-2 and FGF10 double staining were performed to illustrate the intracellular distribution of FGF10. (**F,G**) Comparison of FGF levels in the conditional cultured medium (**F**) or cell lysate (**G**) of primary cortical neurons and astrocytes.

Immunoblotting analysis confirmed the expression of FGF10 in mouse brain tissue (Fig. 1A). FGF10 was also detected in mouse CSF (Fig. 1A), suggesting FGF10 can be released by neural cells in CNS. To explore which type of neural cells is the main source of brain FGF10, we first detected FGF10 expression in mouse brain tissue using double staining immunohistochemistry. FGF10 was mainly co-localized with neuron (Fig. 1B, up panel) but not astrocytes (Fig. 1C). Moreover, enlarged images demonstrated that FGF10 protein is located not only in nucleus, but also in cytoplasm (Fig. 1B, low panel).

We also compared FGF10 levels in the conditional culture medium of primary neurons and astrocytes (Fig. 1D). Double-immunochemistry staining confirmed that FGF10 is located in both nucleus and cytoplasm (Fig. 1E). As shown in Fig. 1F–G, the FGF10 levels in both culture medium and cell lysate of primary neurons were much higher than those in astrocytes. These results suggested that FGF10 is expressed in brain, and neuron may be the main source of brain FGF10.

### Brain FGF10 is upregulated in MCAO model

**Figure 2:**
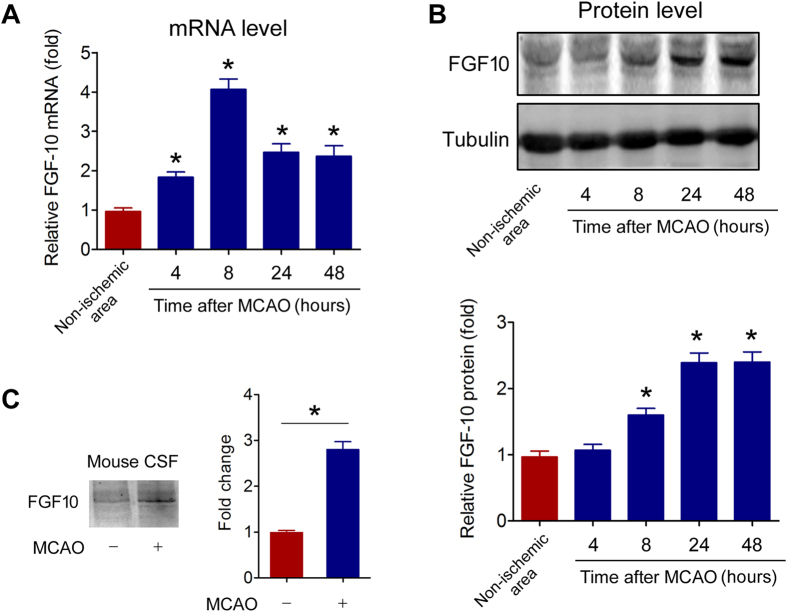
Experimental cerebral ischemia increases brain FGF10 expression and release. (**A,B**) FGF10 mRNA (**A**) and protein (**B**) levels in brain penumbra area were determined by real-time PCR analysis and immunoblotting respectively. *P < 0.05 vs non-ischemic, n = 6 per group. (**C**) FGF10 levels of cerebrospinal fluid (CSF) in MCAO mice and control mice were determined by immunoblotting. *P < 0.05 vs control, n = 3 per group.

Next, we tested the influence of experimental cerebral ischemia on FGF10 mRNA and protein expression. As shown in Fig. 2A, FGF10 mRNA level in brain penumbra area of MCAO mice was significantly higher than that in non-ischemia brain area. The mRNA level of FGF10 reached peak at 8 h post MCAO (~4 folds). Protein expression of FGF10 in brain penumbra area was also enhanced and reached peak at 24 h post MCAO (~2.5 folds, Fig. 2B). Moreover, the FGF10 protein level in CSF was significantly induced by MCAO (Fig. 2C). These results suggest that brain FGF10 is upregulated by experimental cerebral ischemia.

### Exogenous FGF10 treatment ameliorates cerebral ischemic injury and reduces neuronal apoptosis in MCAO model

**Figure 3:**
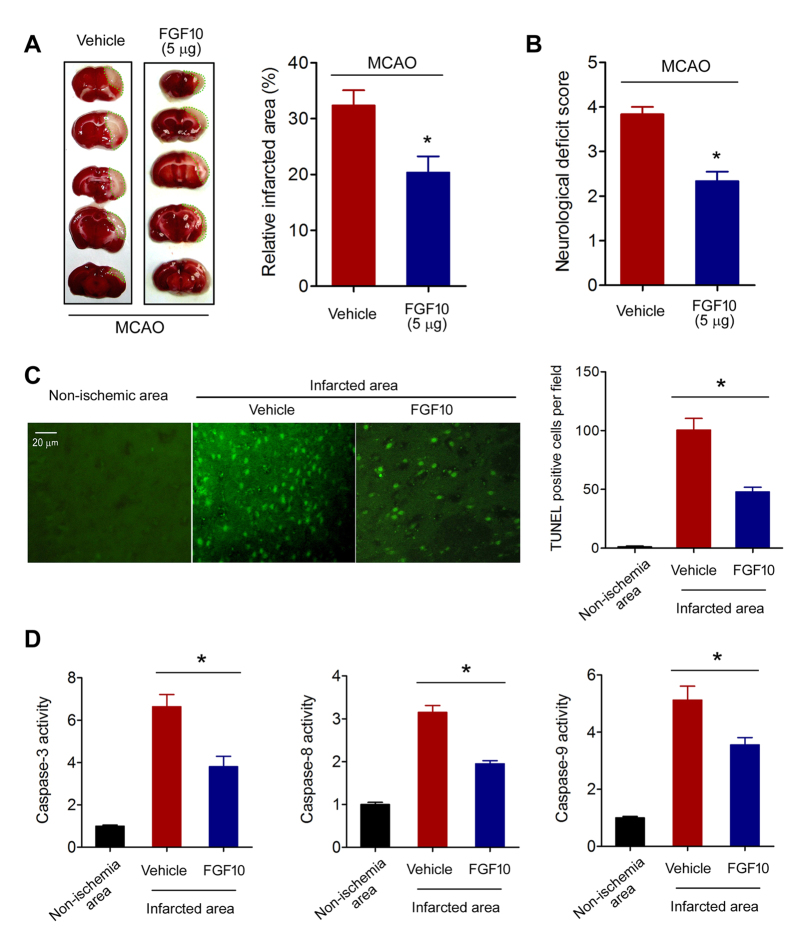
FGF10 treatment reduces ischemic infarcted volume, neurological deficit and neuronal apoptosis in MCAO model. (**A**) Brain infarcted volume was assayed by TTC staining at 24 hours after MCAO. The white area surrounded by green dotted line was considered as infarcted brain tissue. *P < 0.05 vs control, n = 8 per group. (**B**) Neurological deficit score was assessed at 24 hours after MCAO. *P < 0.05 vs control, n = 8 per group. (**C**) Representative images and quantitative analysis of apoptosis using fluorescent TUNEL assay. TUNEL-positive cells (green) were considered as apoptotic cells. *P < 0.05 vs vehicle, n = 6 per group. (**D**) Activities of Caspase-3, Caspase-8 and Caspase-9 in non-ischemic and infarcted brain tissue of MCAO mice were determined. *P < 0.05 vs vehicle, n = 6 per group.

Since brain FGF10 is upregulated by cerebral ischemia, we proposed that the upregulated FGF10 might have important physiopathological roles during cerebral ischemia. Thus, we injected FGF10 (5 μg) into the lateral ventricles using stereotaxic techniques. As shown in Fig. 3A, TTC staining assay demonstrated that FGF10 treatment significantly reduced the brain infarct area in MCAO mice (from ~32% to ~20%, *P* < 0.05). FGF10 treatment also ameliorated the neurological function deficit in MCAO mice (Fig. 3B). TUNEL assay showed that FGF10 treatment decreased the number of apoptotic cells in infarcted area (Fig. 3C). FGF10 treatment suppressed the activities of Caspase-3, Caspase-8 and Caspase-9 (Fig. 3D). These results suggest that FGF10 treatment ameliorates cerebral ischemic injury and reduces neuronal apoptosis in mouse MCAO model.

### FGF10 decreases post-ischemic neuroinflammation in MCAO model

**Figure 4:**
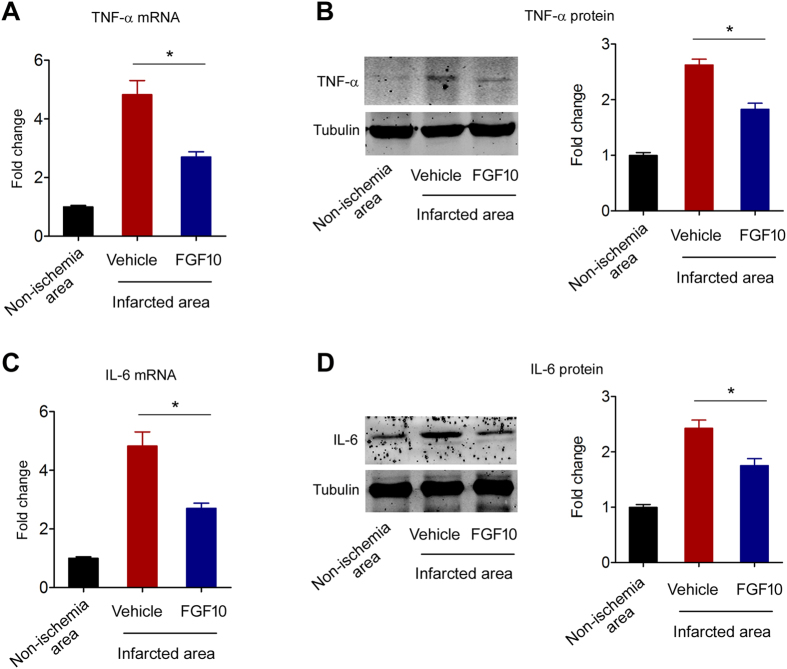
FGF10 treatment decreases post-ischemic neuroinflammation at 24 hours after MCAO. (**A,B**) TNF-α mRNA (**A)** and protein (**B)** expression in non-ischemic and infarcted brain tissue of MCAO mice were determined by real-time PCR analysis and immunoblotting respectively. *P < 0.05 vs vehicle, n = 4 per group. (**C,D**) IL-6 mRNA (**C)** and protein (**D)** expression in non-ischemic and infarcted brain tissue of MCAO mice were determined by real-time PCR analysis and immunoblotting respectively. *P < 0.05 vs vehicle, n = 4 per group.

It is well accepted that post-ischemic neuroinflammation is one of the important mechanisms of ischemic brain injury^19^. MCAO significantly elevated TNF-α mRNA expression, which was partly blocked by FGF10 treatment (Fig. 4A). Also, FGF10 treatment partly inhibited the TNF-α protein upregulation in infarcted brain tissue (Fig. 4B). Similar phenotypes were observed in IL-6. The mRNA and protein levels of IL-6 in infarcted brain tissue were increased, while FGF10 treatment partly depressed these changes (Fig. 4C,D).

### FGF10 treatment inhibits NF-κB signaling pathway and activates PI3K/Akt survival signaling pathway in MCAO model

**Figure 5:**
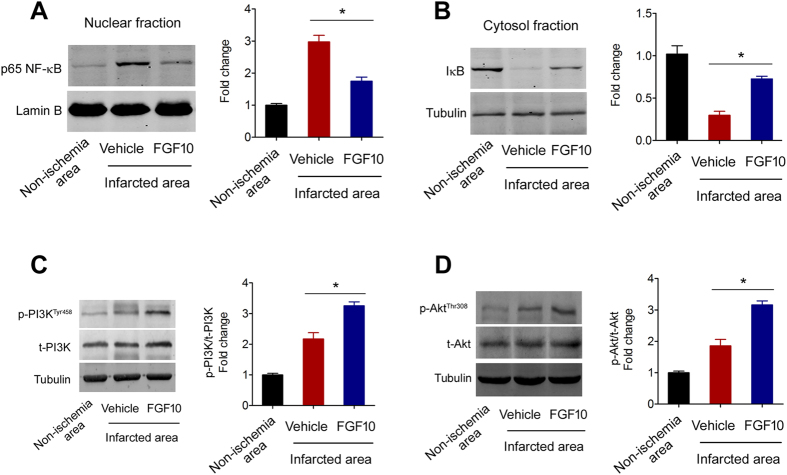
FGF10 treatment inhibits NF-κB signaling pathway and activates PI3K/Akt survival signaling at 24 hours after MCAO. (**A**) Expression of p65 NF-κB in nuclear fractions in non-ischemic and infarcted brain tissue of MCAO mice was determined by immunoblotting. *P < 0.05 vs vehicle, n = 4 per group. (**B**) Expression of IκB in cytosol fractions in non-ischemic and infarcted brain tissue of MCAO mice was determined by immunoblotting. *P < 0.05 vs vehicle, n = 4 per group. (**C**) Phosphorylation of PI3K at Tyr458 site in non-ischemic and infarcted brain tissue was determined by immunoblotting. *P < 0.05 vs vehicle, n = 4 per group. (**D**) Phosphorylation of Akt at Thr308 site in non-ischemic and infarcted brain tissue was determined by immunoblotting. *P < 0.05 vs vehicle, n = 4 per group.

Since NF-κB signaling pathway is the major inflammatory mediator regulating TNF-α and IL-6 production in CNS^20^, we further studied the effects of FGF10 treatment on NF-κB signaling activation in MCAO model. The nuclear NF-κB p65 subunit level (Fig. 5A) whereas the cytosolic IκB expression was lower in infarcted brain tissues compared with non-ischemia brain tissue (Fig. 5B), indicating the NF-κB signaling-dependent neuroinflammation was activated by experimental ischemic stroke. Treatment of FGF10 significantly attenuated the nuclear NF-κB p65 subunit level and upregulated cytosolic IκB level (Fig. 5A,B). These observations indicate that FGF10 suppresses the activation of the NF-κB signaling pathway during ischemic stroke.

We previously showed that in cultured neurons FGF10 induced expression of heme oxygenase-1 (HO-1)^16^, which is a target gene of PI3K/Akt signaling pathway. And PI3K/Akt is one of the most critical pro-survival signaling pathway in ischemia and reperfusion^21^. Therefore, we determined the effect of FGF10 treatment on PI3K/Akt signaling pathway *in vivo*. Centrally FGF10 treatment significantly increased the phosphorylation of PI3K at Tyr 458 site (Fig. 5C) and phosphorylation of Akt at Thr 308 site (Fig. 5D).

### PI3K/Akt signaling pathway is required for the neuroprotection of FGF10

**Figure 6:**
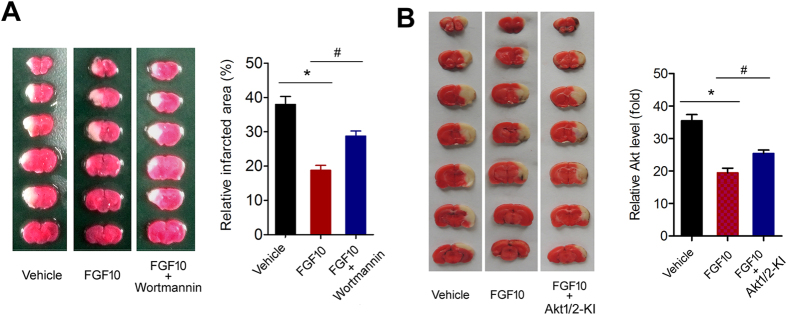
Blockade of PI3K/Akt signaling pathway by wortmannin impairs the neuroprotection of FGF10 at 24 hours after MCAO. (**A**) Effect of wortmannin injection on brain infarcted volume was assayed by TTC staining at 24 hours after MCAO. The white area surrounded was considered as infarcted brain tissue. *P < 0.05 vs control, ^#^P < 0.05 vs FGF10, n = 8 per group. **(B)** Effect of Akt1/2-KI injection on brain infarcted volume was assayed by TTC staining at 24 hours after MCAO. The white area surrounded was considered as infarcted brain tissue. *P < 0.05 vs control, ^#^P < 0.05 vs FGF10, n = 8 per group.

Next we investigated whether the activation of PI3K/Akt signaling is important for the neuroprotection of FGF10. The reduced infarcted brain area by FGF10 treatment was partly reversed by injection of wortmannin, a selective inhibitor of PI3K/Akt signaling (Fig. 6A).

We performed additional experiments using Akt1/2 kinase inhibitor (Akt1/2-KI), another Akt specific inhibitor. Using this specific pharmacological tool, we confirmed that blocking Akt signaling pathway was able to abolish the neuroprotection of recombinant FGF10 (Fig. 6B).

### PI3K/Akt signaling pathway is not essential for the inhibition of inflammation by FGF10

**Figure 7:**
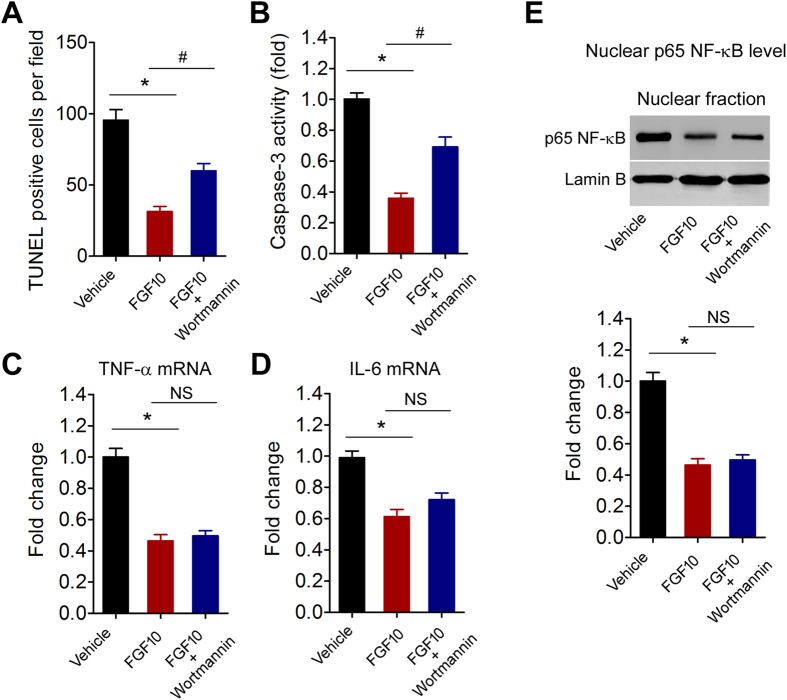
Blockade of PI3K/Akt signaling pathway by wortmannin does not affect the anti-inflammatory action of FGF10 at 24 hours after MCAO. (**A**) Effect of wortmannin injection on apoptosis (TUNEL staining. *P < 0.05 vs control, ^#^P < 0.05 vs FGF10, n = 8 per group. (**B**) Effect of wortmannin injection on Caspase-3 activity in brain tissue. *P < 0.05 vs control, ^#^P < 0.05 vs FGF10, n = 8 per group. (**C,D**) Effect of wortmannin injection on mRNA levels of TNF-α (**C**) and IL-6 (**D**) in infarcted brain tissue. *P < 0.05 vs control, n = 8 per group. NS, no significance. (**E**) Effect of wortmannin injection on nuclear p65 NF-κB expression in infarcted brain tissue. *P < 0.05 vs control, n = 8 per group. NS, no significance.

Further, wortmannin injection partly abolished the decreased apoptosis (Fig. 7A) and Caspase-3 activity (Fig. 7B) by FGF10 treatment. However, wortmannin did not impair the inhibitory effect of FGF10 on TNF-α and IL-6 production (Fig. 7C,D). Wortmannin also did not affect the inhibitory effect of FGF10 on nuclear NF-κB p65 subunit level (Fig. 6E). These results suggest that activation of PI3K/Akt signaling pathway by FGF10 is required for the neuroprotection of FGF10, but may be not essential for the anti-inflammatory action of FGF10.

## Discussion

**Figure 8:**
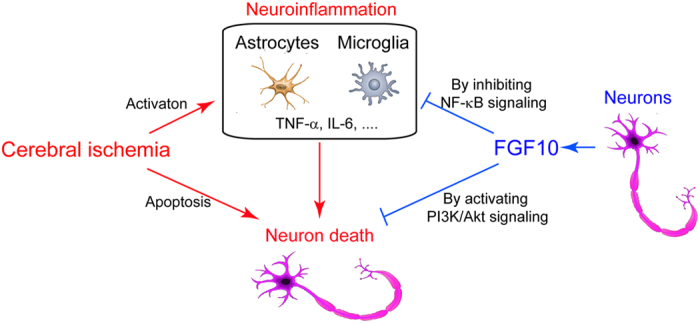
A proposed framework for the neuroprotection of neuron-derived FGF10 against cerebral ischemic injury by inhibiting NF-κB-dependent neuroinflammation and activating PI3K/Akt survival signaling pathway.

In the present study, we provide the first evidence that neuron-derived FGF10 ameliorates cerebral ischemia injury *in vivo* (Fig. 8). Firstly, we found that brain FGF10 was upregulated by ischemic insult and intracranial FGF10 administration successfully reduced brain infarcted volume. Secondly, FGF10 treatment *in vivo* decreased neuronal apoptosis and inhibited NF-κB signaling activation during ischemic stroke and thereby neuroinflammation (TNF-α and IL-6 production). Thirdly, FGF10 treatment activated phosphorylation of PI3K/Akt signaling pathway. At last, blockade of PI3K/Akt compromised the neuroprotection of FGF10 but did not diminish the anti-inflammatory effect of FGF10.

The first interesting finding of this study is that we found FGF10 is a neuron-derived paracrine factor and can be induced by ischemic insult. Previous studies have showed FGF10 expression in brain^22^,^23^,^24^. In the present study, we found that FGF10 was detected in CSF. Moreover, we observed that neurons, but not astrocytes, may be the main source of secreted FGF10. These results are in line with a very recent report^25^ and suggest that FGF10 is a neuronal secretory factor. We propose that neurons, but not astrocytes, may be the major source of cerebral FGF10 according to our results. Additionally, both brain FGF10 expression and FGF10 level in CSF were induced in MCAO mice. In view of that our previous results have revealed the potential neuroprotection of FGF10 in cultured neuron model (*in vitro*), we considered that the upregulated brain FGF10 level by cerebral ischemia might affect the outcome of ischemic stroke *in vivo*.

To further answer this question, we directly injected recombinant FGF10 into lateral cerebroventricle in MCAO mice. Our previous study suggested that FGF10 has neuroprotection in neuron OGD model^16^. However, the pathophysiological changes in brain are far more complex than that in neurons. So we further studied FGF10 in MCAO model. As expected, FGF10 reduced the infarcted area and ameliorates neurological deficit. Moreover, ischemia-induced neuronal apoptosis was also inhibited by FGF10 treatment. In agreement with our previous findings showing the anti-apoptotic activity of FGF10 in cultured neurons^16^, the present results provide the first *in vivo* evidence supporting the neuroprotection of FGF10 in brain. This result is in line with previous studies showing that FGF1 and FGF2 protects against ischemic brain injury^26^,^27^. Since neuroprotection *in vivo* may be resulted by multiple parameters that are not confined to anti-apoptosis activity, we determined post-ischemic cerebral inflammation, another critical factor contributing neuronal injury after ischemic stroke. Interestingly, we detected significant decreases of TNF-α and IL-6 mRNA and protein expression in brain of FGF10-treated mice. Additionally, nuclear p65 NF-κB level was reduced, while cytoplasmic IκB level was increased in brain of FGF10-treated mice. The brain responds to ischemic damage with an acute and prolonged inflammatory activation which are characterized by both rapid activation of resident microglia and subsequent infiltration of various types of inflammatory cells (including neutrophils, different subtypes of T cells, monocyte/macrophages, and other cells) into the ischemic area^28^. The proinflammatory factors such as TNF-α and IL-6 are released by activated microglia and other inflammatory cells^28^. In patients with ischemic stroke, IL-6 levels in plasma and cerebrospinal fluid (CSF) are associated with early clinical deterioration, indicating that proinflammatory cytokines and early neurological worsening in ischemic stroke^29^. The attenuated proinflammatory cytokine level by FGF10 treatment implies the possible effects of FGF10 on microglia and other inflammatory cells. Although there is no previous study about the effect of FGF10 on microglia or other inflammatory cells, we noted that both direct NF-κB activation and inflammatory cytokines reduced FGF10 expression in bronchopulmonary dysplasia^30^, which suggests a potential causal association between FGF10 and inflammation. However, the exact role of FGF10 in post-ischemic inflammation, especially in the activation of astrocytes and microglia, may need more studies in future.

PI3K/Akt signaling is a critical growth and survival pathway in many biological processes, especially on the process of early ischemia/reperfusion injury as we previously described^31^. So far, no negative effects of PI3K/Akt signaling on the ischemic stroke have been found^21^. We previously showed that FGF10 protects neuron against OGD injury through inducing HO-1^16^. In the present study, we found that FGF10 treatment activated phosphorylation of PI3K/Akt in brain tissue, suggesting that the activation of Akt by FGF10 may account for the upregulation of HO-1 in neuron. In addition, blockade of PI3K/Akt signaling by wortmannin, a selective chemical inhibitor^32^, partially abrogated the anti-apoptotic activity of FGF10. However, wortmannin failed to attenuate the anti-inflammatory action of FGF10. These results indicated that activation of PI3K/Akt signaling pathway is required for the neuroprotection of FGF10 but not for the anti-inflammation action of FGF10. The inhibition on NF-κB-dependent neuroinflammation and activation of PI3K/Akt signaling pathway by FGF10 might be two independent molecular mechanisms for the neuroprotection of FGF10 *in vivo*. The activation on Akt signaling by FGF10 was observed in hepatocytes^33^ and endothelial cells^34^, but not in lung mesenchymal cultures^35^ and human endometrial carcinoma cells^36^. This cell type-dependent activation on Akt signaling by FGF10 may attribute to the presence or absence of FGFR2B, the receptor of FGF10^11^,^12^,^13^. Previously, a large number studies have showed that Akt activation positively controls NF-κB-dependent transcription, whereas blocking PI3K/Akt signaling leads to a marked reduction of constitutive NF-κB activity^37^,^38^,^39^,^40^,^41^. In our study, we found that FGF-10 activated Akt signaling pathway, which seemed to be able to result in an enhanced NF-κB activity under ischemic stress. Nevertheless, immunoblotting assay demonstrated that FGF10 inhibited NF-κB activity in our experiments (Fig. 5), and blocking Akt by wortmannin did not affect the inhibitory effect of FGF10 on nuclear NF-κB p65 subunit level in cerebral ischemic model (Fig. 7). These results clearly point out that the inhibitory effect of FGF10 on NF-κB activity is not mediated by Akt. We did not know how FGF10 modulates NF-κB indeed. In addition, we are still trying to find which signaling pathway contributes to the suppressed NF-κB activity upon FGF10 treatment. We think this issue may need further clarification with detailed works as it raises an important question about the complexity of FGF10-induced biological functions in ischemic stroke.

As an extracellular protein, the half-life time of FGF10 is critical for its potential application in future clinics. Buchtova *et al.* recently showed that FGFs, including FGF1, FGF3, FGF4, FGF6, FGF8, FGF9, FGF10, FGF16, FGF17, FGF18, FGF20, and FGF22, exist as unstable proteins with half-life times as 0.5 ~ 4 hours in cell-free medium; however, exogenous heparin binding rescued stabilization of these FGFs^42^. In our opinion, this feature of FGF10 would not hamper its therapeutic value in stroke because the time window for stroke treatment is 3.5–4 hours^1^ and the intracellular signaling cascade triggered by a bolus FGF10 treatment could still exert biological functions in this period. In this study, we detected inflammation, apoptosis, and PI3K pathway in infarcted area and detected protein and mRNA changes of FGF10 in penumbra. We considered that the apoptosis and inflammation are important processes occurred mainly in died or dying neural cells. These phenotypes would be more pronounced in the infracted areas. Thus, we detected inflammation and apoptosis, as well as the PI3K/Akt signaling pathway, which might regulate NF-κB transduction to control inflammation and apoptosis in infarcted area. By contrast, the upregulation and release of FGF10 are intracellular adaptive responses which require living neural cells. In infracted areas, living neural cells are sparse. So we detected the upregulation and release of FGF10 in penumbra.

Most of FGFs are likely to be localized predominantly in cytoplasmic region and cell surface. However, the nuclear localization of a number of FGFs has been reported in various cell lines and tissues. The FGF1 and FGF2 (also known as basic FGF [bFGF]) are two most studied members of FGFs. These two FGFs have been reported to locate in both nuclei and cytoplasm^43^. Moreover, there are many researches regarding the molecular mechanisms of their nuclear translocation and export. The nuclear translocation has been demonstrated to be associated with neurotrophic activity^44^, tumor^45^,^46^, ischemic injury^47^ and other biological functions^48^. In addition, Bryant DM *et al.* have reviewed the biological significance of nuclear translocation of FGFs^43^. We detected FGF10 in both cytoplasm and nuclei in brain tissues, which indicates that neural FGF10 may also have nuclear translocation process. Moreover, the possible nuclear translocation process of FGF10 may play an important role in biological function of FGF10. However, this interesting question needs further detailed investigation.

In conclusion, we demonstrate that neuron-derived FGF10 ameliorates cerebral ischemia injury via inhibiting NF-κB-dependent neuroinflammation and activating PI3K/Akt survival signaling pathway in mice. These findings have implications for understanding the pathophysiology of ischemic stroke and may open new directions focusing on the therapeutic value of FGF10 in the treatment of ischemic stroke.

## Materials and Methods

### Reagents

Recombinant FGF10 (catalogue: 345-FG-025) was purchased from R&D Systems (Minneapolis, MN). Antibodies against FGF10 (ab115825), MAP-2 (ab5392), GFAP (ab7260), tubulin (ab179513) and t-PI3K (ab22653) were purchased from Abcam (Cambridge, MA). Antibodies against p65 nuclear factor-κB (NF-κB, sc-372), IκB (sc-371), TNF-α (sc1351) and IL-6 (sc-1265) were purchased from Santa Cruz Biotechnology, Inc. (Santa Cruz, CA). Antibody against p-Akt (2965), t-Akt (9272) and p-PI3K (4288) was purchased from Cell Signaling Biotechnology (Danvers, MA). Fluorescent terminal deoxynucleotidyl transferase dUTP nick end labeling (TUNEL) assay kit was purchased from Promega (Medison, WI). Caspase-3, −8 and −9 activity colorimetric kits were purchased from Abcam. 2,3,5-triphenyltetrazolium chloride (TTC), wortmannin and Akt1/2-KI were purchased from Sigma Chemical Company (St. Louis, MO)

### Animals

Male 8–12 weeks old C57BL/6 or CD1 mice were supplied by the Shanghai Sino-British Sippr BK Lab Animal Co., Ltd. Animals were housed in a facility with controlled temperature (23 ± 2 °C) and lighting (08: 00 to 20: 00 h), with free access to tap water. All animal experiments were approved by the Animal Ethics Committee of Second Military Medical University and were performed in compliance with the Guide for Care and Use of Laboratory Animals published by the US National Institutes of Health (NIH publication no. 85–23, revised 1996).

### Cerebrospinal fluid collection

CSF was from male CD1 mice (4 months) anesthetized with pentobarbital sodium (60 mg/kg, ip) as described previously^49^. We collected 40 μl CSF from 8 mice and concentrated it to 8 μl using Amicon® Ultra Centrifugal Filters (EMD Millipore, Billerica, MA) for immunoblotting.

### MCAO

MCAO surgery in mice was performed as described previously^50^,^51^. Briefly, mice were anesthetized with chloral hydrate (400 mg/kg, i.p.) and the core temperature (rectum) was maintained at 36.5 °C–37.5 °C using a homeothermic heating pad (CWE Inc., Ardmore, PA) throughout the surgery. Cerebral focal ischemia was produced by intraluminal occlusion of the left middle cerebral artery using a silicone rubber-coated nylon monofilament. The cerebral blood flow was reduced by more than 85% using a laser Doppler flowmeter (VMS^TM^-LDF1; Moor Instruments, Axminster, UK). Two hours after MCAO, the occluding filament was withdrawn to allow reperfusion. Penumbra is an established concept in stroke, defined as a region of hypoperfused, metabolically active tissue surrounding the ischemic core^52^. The non-ischemic area is defined as the contralateral brain tissue.

### Neurological deficit score and TTC staining

Mice were examined for neurological deficit using a 5-point scale^50^,^53^. Animals with normal motor function were scored as 0, flexion of the contralateral torso and forearm on lifting the animal by the tail as 1, circling to the contralateral side but normal posture at rest as 2, leaning to the contralateral side as 3, and no spontaneous motor activity as 4. For TTC staining, brain was removed swiftly from mice being deeply anesthetized (400 mg/kg, i.p.) and sacrificed and was cut into slices with brain-cutting matrix (ASI Instruments, Warren, MI). Then the slices were bathed in the TTC solution at 37 °C for 30 minutes. The TTC-stained brain slices were photographed with a digital camera. Infarction volume was the sum of all lesion areas multiplied by slice thickness with Image J software (National Institutes of Health, USA)^50^.

### Drug treatment *in vivo*

Anesthetized mice (chloral hydrate, 400 mg/kg, i.p.) were fixed in a stereotactic frame (ASI Instruments, Houston, TX) and were injected with FGF10 (5 μg, dissolved in 4 μl saline) or vehicle (saline) into the left lateral cerebroventricle (1 mm posterior, 1.0 mm lateral, and 2.0 mm ventral to the bregma) over 10 minutes as described previously^50^. During the 10 minutes of i.c.v. infusion, a homeothermic heating blanket was used to maintain the core temperature (rectum) at 37 °C. For experiments with PI3K/Akt signaling blockade, wortmannin (0.2 μM, 1 μl) or Akt1/2-KI (0.5 μM, 1 μl) dissolved in saline was injected into the left lateral cerebroventricle after FGF10 injection. The MCAO model was performed at 30 minutes after FGF10 infusion. At 24 hours post MCAO, the non-ischemia brain tissue and infarcted brain tissues were dissected carefully and frozen at −80 °C for subsequent molecular assays.

### Cell culture and conditional culture medium

Primary mouse cortical neurons and astrocytes were used in this study. For primary neuron culture, the dissociated cortical cells were added to poly-L-lysine-coated culture plates and maintained in Neurobasal medium supplemented with 2% B27^54^. Glial growth was suppressed by addition of 5-fluoro-2-deoxyuridine and uridine (10 μM), yielding cultured cells with >95% neurons. After 6 days *in vitro*, the neurons were used for experiments. For primary astrocytes culture, the dissociated cortical cells were added to poly-L-lysine-coated culture plates and maintained in DMEM/F12 medium supplemented with 10% FBS. After 12 days *in vitro*, the astrocytes were used. For collection of conditional culture medium, the neurons and astrocytes were cultured for 3 days without changing the medium. Conditional medium (2 ml) was collected and concentrated to ~50 μl using Microcon-10 kDa Centrifugal Filter Unit (Millipore, Bedford, MA) as described previously^55^.

### Real-time quantitative PCR

Real-time quantitative PCR was performed as described previously^56^,^57^. Total RNA for real-time quantitative RT-PCR was isolated from tissues using RNAiso Reagent (TaKaRa, Tokyo, Japan) and reverse transcripted into the single strand cDNA. Primers were designed using the Primer Express software version 1.5 (Applied Biosystems, Foster City, CA, USA). The primers for TNF-α are as follows: sense, 5′-TTCTGTCTACTGAACTTCGGGGTGATCGGTCC-3′; antisense, 5′-GTATGAGATAGCAAATCGGCTGACGGTGTGGG-3′. The primers for IL-6 are as follows: sense, 5′-ATGGATGCTACCAAACTGGAT-3′ and antisense, 5′-TGAAGGACTCTGGCTTTGTCT-3′. Gene expression of GAPDH was used for control. The primers for GAPDH are as follows: forward, 5′-TCTGACGTGCCGCCTGGAG-3′; reverse, 5′-TCGCAGGAGACAACCTGGTC-3′. Quantification of mRNA was performed using the ABI Prism 7500 (Applied Biosystems) with PrimeScript™ RT-PCR Kit (TaKaRa, Tokyo, Japan).

### Immunoblotting

Immunoblotting analysis was performed as described previously^58^. Brain tissues were lysed with RIPA buffer with protease inhibitors. Protein samples were separated in 12% SDS-PAGE, and transferred onto PVDF membranes at 100V for 1~2 h. After blocking by 5% evaporated milk, membranes were incubated with primary antibodies diluted in TBS containing 1% w/v bovine serum albumin and followed by HRP-labeled secondary antibody^59^. The signal of immunoblotting was detected using the enhanced chemiluminescence system.

### Immunofluorescence and immunohistochemistry

Immunofluorescence and immunohistochemistry were performed as described previously^16^,^57^. Cultured cells or brain sections were fixed in 4% paraformaldehyde, blocked by 8% normal goat serum, and incubated in specific primary antibodies as follows: NeuN (1:500), GFAP (1:1000), MAP-2 (1:400) and FGF10 (1:500). After being washed three times by PBS, the sections and cells were incubated with Alexa 488-conjugated or Alexa 555-conjugated secondary antibodies. Images were obtained by fluorescence microscope (IX-71, Olympus, Tokyo, Japan) with a digital camera (Olympus).

### TUNEL assay

Immunofluorescence TUNEL assay was performed according to the instructions of manufacturer (Roche, Penzberg, Germany) as described previously^53^,^60^. Images were obtained by fluorescence microscope (IX-71, Olympus, Tokyo, Japan) with a digital camera (Olympus).

### Caspases activity

Activities of Caspase-3, 8 and 9 were assessed as described in our previous study^16^. Briefly, brain tissues were lysed and then performed according to the manufacturer’s instruction. Samples were read at 405 nm in a microtiter plate reader. Fold-increase in Caspase-3, −8 and −9 activities were determined by comparing these results with the level of control (non-ischemic brain tissue).

### Statistical analysis

Data are expressed as mean ± SEM. Differences were evaluated by two-tailed Student’s t test or ANOVA followed by Tukey’s post-hoc test with GraphPad Prism 5.0 version. Statistical significance was set at P < 0.05.

## Additional Information

**How to cite this article**: Li, Y.-H. *et al.* Neuron-derived FGF10 ameliorates cerebral ischemia injury via inhibiting NF-κB-dependent neuroinflammation and activating PI3K/Akt survival signaling pathway in mice. *Sci. Rep.* **6**, 19869; doi: 10.1038/srep19869 (2016).
